# Somatosensory evoked potentials and high-frequency oscillations after transcranial static magnetic stimulation over the primary somatosensory cortex

**DOI:** 10.1038/s41598-026-38767-2

**Published:** 2026-02-05

**Authors:** Yuki Tanaka, Aoki Takahashi, Riku Ishizaka, Kodai Minami, Taisei Miyazaki, Kenta Oguma, Nodoka Shimizume, Isamu Ozaki, Tatsunori Watanabe

**Affiliations:** 1 Graduate School of Health Sciences, Aomori University of Health and Welfare, Aomori, Japan; 2 Department of Rehabilitation Sciences, Hirosaki University of Health and Welfare, Hirosaki, Aomori Japan; 3 Department of Advanced Technology in Medicine, Biomedical Engineering Laboratory, Institute of New Industry Incubation, Institute of Science Tokyo, Tokyo, Japan; 4 Waseda Institute for Sport Sciences, Waseda University, Tokorozawa, Saitama Japan; 5 Graduate School of Health Sciences, Aomori University of Health and Welfare, 58-1 Mase, Hamadate, Aomori, 030-8505 Japan

**Keywords:** Transcranial static magnetic stimulation, Event-related potentials, High-frequency oscillations, Non-invasive brain stimulation, Plasticity, Neuroscience, Physiology

## Abstract

Transcranial static magnetic field stimulation (tSMS) is a non-invasive brain stimulation technique known to reduce cortical excitability. Although its effectiveness has been demonstrated in various cortical regions, it remains unclear whether somatosensory processing is influenced by this stimulation. The purpose of this study was to investigate whether tSMS over the primary somatosensory cortex (S1) modulates somatosensory evoked potentials (SEPs) and high-frequency oscillations superimposed on them (somatosensory HFOs). In a randomized crossover study, twenty healthy young adults received tSMS or sham stimulation over the left S1 (corresponding to C3 of the international 10–20 system) for 20 min. SEPs were recorded in response to right median nerve stimulation before, immediately after, and 20 min after stimulation. Somatosensory HFOs were extracted by applying a 400–800 Hz band-pass filter to SEP waveforms and separated into early and late components relative to the N20 peak latency. TSMS over the left S1 significantly reduced the amplitude of early HFOs 20 min after stimulation, whereas late HFOs remained unchanged. No effects of tSMS were observed on the N20 component of standard SEPs, which reflects neural activity in area 3b of the S1. Early and late HFOs are thought to reflect the action potentials of thalamocortical fibers and the activity of GABAergic interneurons, respectively. These findings therefore suggest that tSMS over the S1 selectively attenuates thalamocortical input while exerting no effect on GABAergic interneuron activity.

**Supplementary Information:**

The online version contains supplementary material available at 10.1038/s41598-026-38767-2.

## Introduction

Transcranial static magnetic field stimulation (tSMS) is a novel, non-invasive brain stimulation (NIBS) technique that reduces cortical excitability by placing a small neodymium (NdFeB) magnet over the scalp^1,2^. It has gained attention as a safe and easily administered method for modulating brain activity without the use of electrical current^3^. Previous studies have demonstrated that tSMS can modulate neural activity across a wide range of cortical regions^4–11^. In particular, the effects of tSMS over the primary motor cortex (M1) have been consistently demonstrated across multiple studies^1,12–14^. This accumulating evidence has encouraged intervention studies in clinical populations, such as individuals with Parkinson’s disease, amyotrophic lateral sclerosis, and stroke^15–17^. However, findings regarding the effects of tSMS on the primary somatosensory cortex (S1) have been inconsistent and thus remain to be clarified.

Somatosensory evoked potentials (SEPs) are cortical responses elicited by peripheral nerve electrical stimulation, reflecting the transmission of sensory signals via the dorsal columns and thalamus to the S1^18^. Clinically, SEPs are used to detect lesions in peripheral nerves and cerebral hemispheres^19^. A previous study reported that tSMS over the S1 attenuated the amplitude of the N20 component of the SEP^20^, which reflects neural activity in area 3b of the S1^21^. However, a subsequent study employing a larger sample size failed to demonstrate a significant effect of tSMS on the N20 component, even with a longer stimulation duration (extended from 10/15 to 20 min)^22^, in which the number of averaged SEP responses was more than twice that of the previous study^20^, resulting in an improved signal-to-noise ratio. Thus, the effects of tSMS on SEPs remain inconclusive. On the other hand, high‑pass filtering of SEP waveforms enables the extraction of high‑frequency oscillations in the 400–800 Hz range, which are superimposed on the N20 component^23^. In the present study, these oscillations recorded from the somatosensory cortex are referred to as *somatosensory HFOs* to distinguish them from physiological ripples observed in the hippocampus and parahippocampal gyrus, as well as fast ripples recorded from epileptogenic zones. Somatosensory HFOs can be further classified into early HFOs (eHFOs) and late HFOs (lHFOs), based on their timing relative to the N20 peak^24^. EHFOs are thought to reflect the action potentials of thalamocortical fibers, whereas lHFO are attributed to the activity of GABAergic interneurons within area 3b of the S1^23^. Importantly, inhibitory NIBS techniques, including low-frequency repetitive transcranial magnetic stimulation (rTMS), cathodal transcranial direct current stimulation (tDCS), and continuous theta burst stimulation (cTBS), have been shown to modulate somatosensory HFOs when applied to the S1^25–27^. However, the effects of tSMS on somatosensory HFOs have not yet been investigated. Given the inconsistent findings regarding the impact of tSMS on GABAergic interneuron activity^12,28–30^, examining changes in lHFOs following tSMS over the S1 may offer new insights into its underlying mechanisms of action.

The purpose of this study was to investigate the effects of tSMS over the S1 on the N20 component and somatosensory HFOs. We hypothesized that tSMS would reduce the amplitudes of the N20 as well as both early and late HFOs. By comparing the effects of tSMS on N20 and somatosensory HFOs, we aimed to delineate whether these components are modulated in a similar or distinct manner, thereby enhancing our understanding of how tSMS influences somatosensory cortical activity.

## Materials and methods

### Participants

Sample size was calculated using G*Power with an effect size of 0.25, an alpha of 0.05, and a statistical power of 0.80, which indicated a required sample size of nineteen participants. Ultimately, twenty healthy young adults (mean age ± SD = 22.4 ± 2.9, aged 18–26 years, thirteen males and seven females) participated in this study. Participants were screened to exclude any history of neurological, psychiatric, cognitive, orthopedic, or cardiopulmonary disorders that could influence the study. All participants gave written informed consent after receiving a full explanation of the study procedures. The research protocol was approved by the Ethics Committee of Aomori University of Health and Welfare (approval number: 240646) and conformed to the Declaration of Helsinki. This study was registered in the University hospital Medical Information Network Clinical Trials Registry (UMIN-CTR) on May 10, 2024 (trial ID: UMIN000054358), and adhered to the CONSORT guidelines (Supplementary file).

### Experimental procedure

**Fig. 1:**
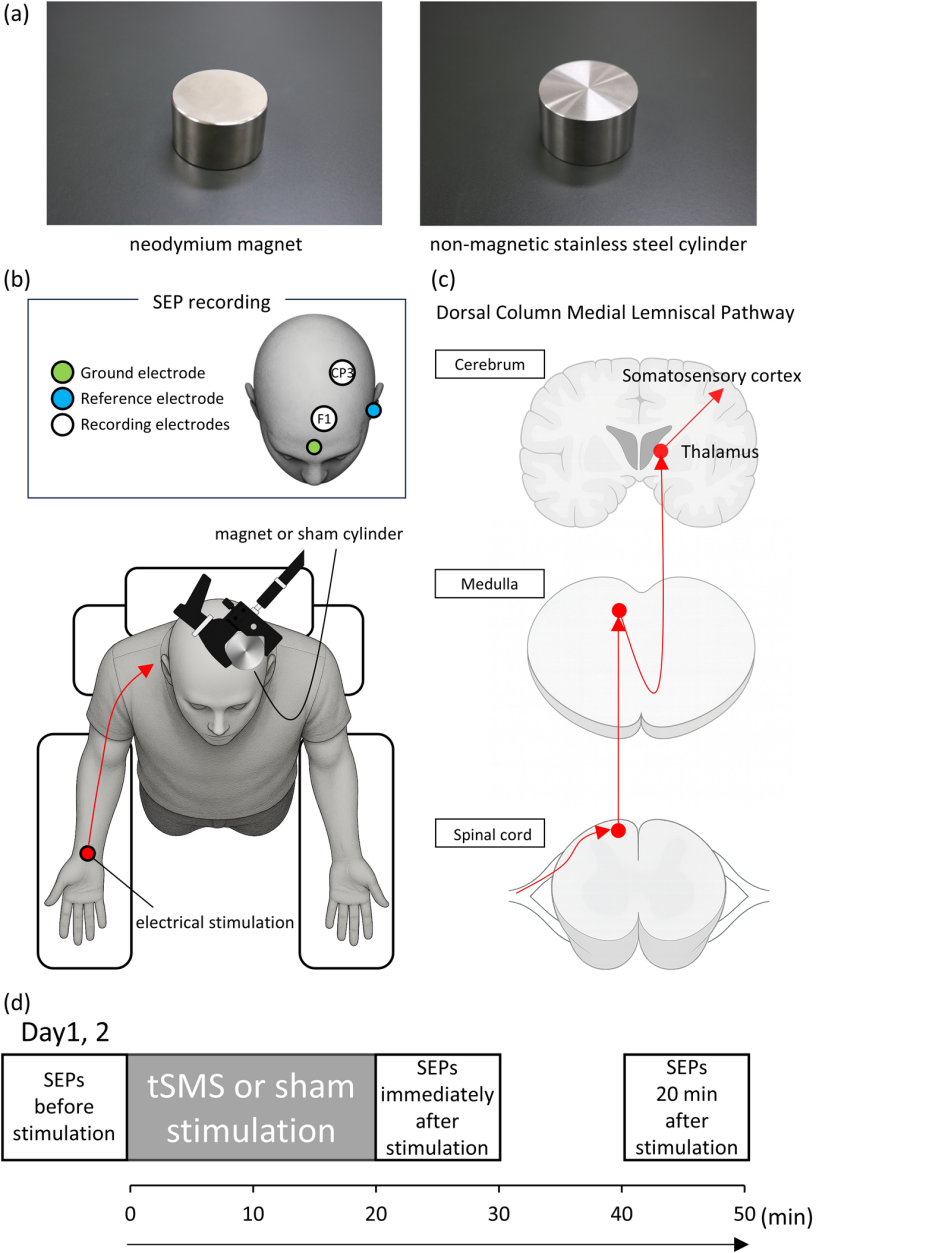
Experimental apparatus and procedure (**a**) A cylindrical neodymium magnet (NdFeB; diameter: 50 mm; height: 30 mm; surface magnetic flux density: 534 mT; maximum energy density: 49 MGOe; strength: 862 N) was used for transcranial static magnetic stimulation (tSMS). An identically sized, weighted, and visually matched non-magnetic stainless steel cylinder was used for sham stimulation. (**b**) Participants were seated in a comfortable reclining chair equipped with a headrest, with their arms resting on arm tables. The NdFeB magnet or sham cylinder was placed over the primary somatosensory cortex (C3 of the international 10–20 system) using an adjustable-arm light stand. Somatosensory evoked potentials (SEPs) were elicited by electrical stimulation of the right median nerve. The N20 and P25 components of SEP were evaluated at CP3, and high-frequency oscillations (somatosensory HFOs) were evaluated from the CP3-F1 trace. (**c**) Schematic illustration of the anatomical pathway assessed by median nerve SEPs (dorsal column medial lemniscal pathway). (**d**) SEPs were recorded before, immediately after, and 20 min after tSMS or sham stimulation.

Participants were seated in a comfortable reclining chair equipped with a headrest in a laboratory setting. In the tSMS condition, a cylindrical neodymium magnet (NdFeB; diameter: 50 mm; height: 30 mm; surface magnetic flux density: 534 mT; maximum energy density: 49 MGOe; strength: 862 N) was used (NeoMag Co., Ltd., Ichikawa, Japan). In the sham condition, a non-magnetic stainless steel cylinder matched in shape, weight, and appearance was applied (Fig. 1a). The device (tSMS or sham) was positioned over the left S1, corresponding to the C3 of the international 10–20 system, and was held in place for 20 min using a movable arm-type light stand (C-stand, Avenger, Cassola, Italy) (Fig. 1b). SEPs elicited by electrical stimulation of the right median nerve were recorded at three time points: before, immediately after, and 20 min after stimulation (Fig. 1b–d). In this single-blind randomized crossover design, the order of stimulation conditions was randomized across participants using the random number generator method. To minimize carryover effects, the two sessions were conducted on separate days with an interval of at least one week.

### SEP recording

SEPs were recorded at a sampling rate of 10,000 Hz with a band-pass filter of 1–2000 Hz using a signal processor (Neuropack MEB-2300 system; Nihon-Kohden, Tokyo, Japan). Electrodes were placed at F1, F3, F5, FC1, FC3, FC5, C1, C3, C5, CP1, CP3, and CP5 according to the international 10–20 system, similar to a previous study^31^. The ground electrode was placed on the forehead, and the reference electrode was attached to the left earlobe. Electrode impedance was maintained below 5 kΩ. Brief electrical pulses (0.2 ms) were delivered to the right median nerve at a frequency of 4.7 Hz, with intensity adjusted to elicit a slight muscle twitch in the thenar muscles. A total of 2,500 responses were recorded at each time point (before, immediately after, and 20 min after stimulation), with trials exceeding 100 µV automatically rejected to exclude those contaminated by artifacts.

### Data analysis

**Fig. 2:**
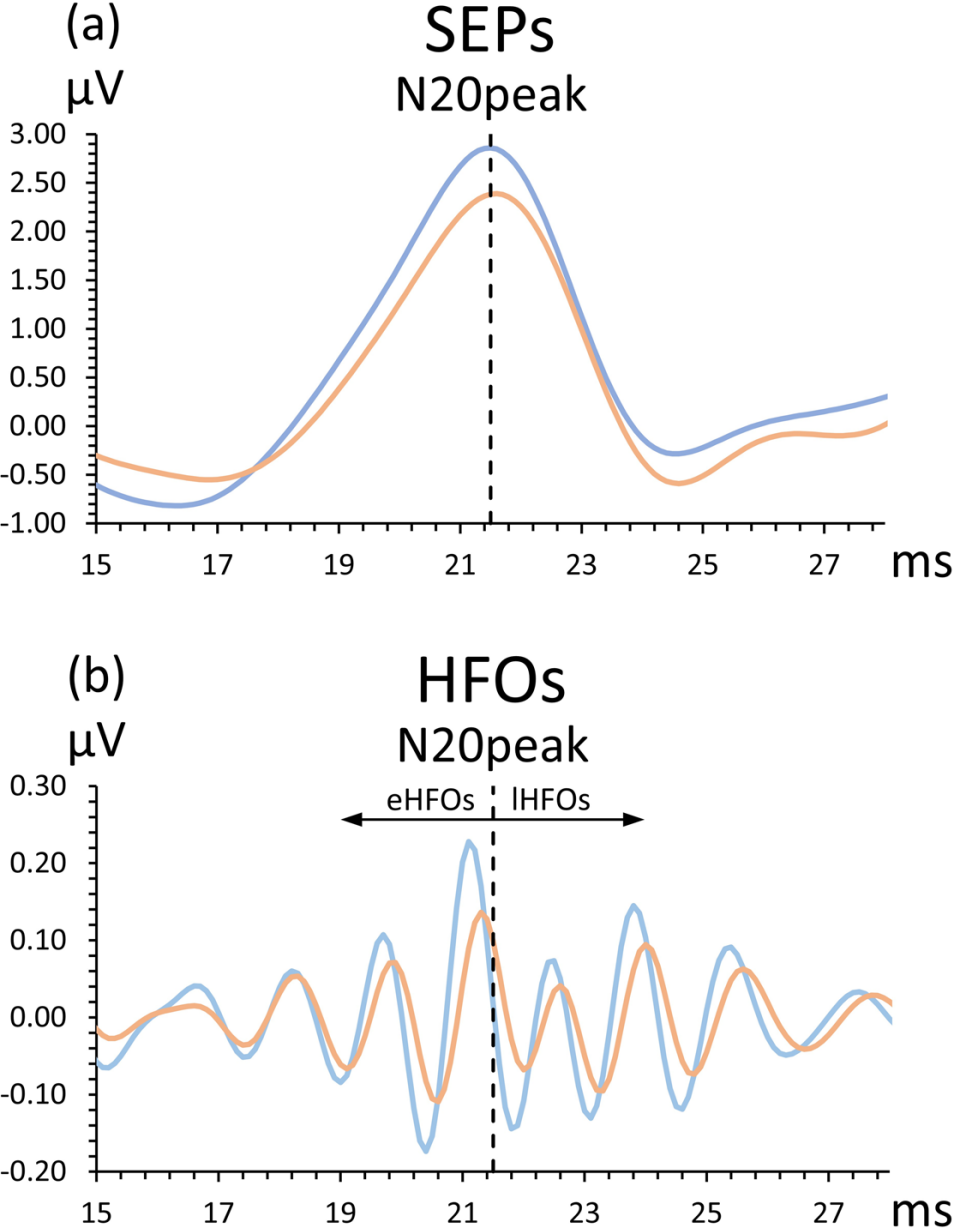
Representative waveforms (**a**) Low-frequency somatosensory evoked potentials (SEPs) and (**b**) high-frequency oscillations (somatosensory HFOs). Somatosensory HFOs were divided into early HFOs and late HFOs based on the N20 peak. Blue lines represent waveforms recorded before tSMS, whereas orange lines represent those recorded after tSMS.

A total of 2,500 responses were averaged for each participant. Low-frequency components were analyzed from SEPs recorded at CP3, after applying a 300 Hz low-pass filter (Fig. 2a). We measured the onset-to-peak amplitude of the N20 and the peak-to-peak amplitude from the N20 peak to the P25 peak (P25). Although the amplitude of P25 is known to attenuate as stimulation frequency increases (e.g., at 4.7 Hz), this variable was included because its amplitude varies with that of lHFOs.

A CP3-F1 trace filtered at 400–800 Hz was obtained to extract the somatosensory HFOs, after confirming that the frontal and contralateral parietal waves exhibited an out-of-phase relationship^31^. The somatosensory HFOs were divided into two components: eHFOs (from the N20 onset to the N20 peak), and lHFOs (from the N20 peak to the P25 peak) (Fig. 2b). The somatosensory HFOs were then rectified, and the area of the waves was measured for both eHFOs and lHFOs.

### Statistical analysis

Statistical analyses were performed using two-way repeated-measures ANOVA with stimulation (tSMS vs. Sham) and time (pre-, immediately post-, and 20 min post-stimulation) as within-subject factors. Data normality was assessed with the Shapiro–Wilk test, and non-normal data were log-transformed prior to ANOVA. Sphericity was assessed using Mauchly’s test, and when violated, the Greenhouse–Geisser correction was applied. Post hoc comparisons were conducted using Bonferroni correction for multiple testing. Statistical significance was set at α = 0.05.

## Results

### N20 amplitude

**Fig. 3:**
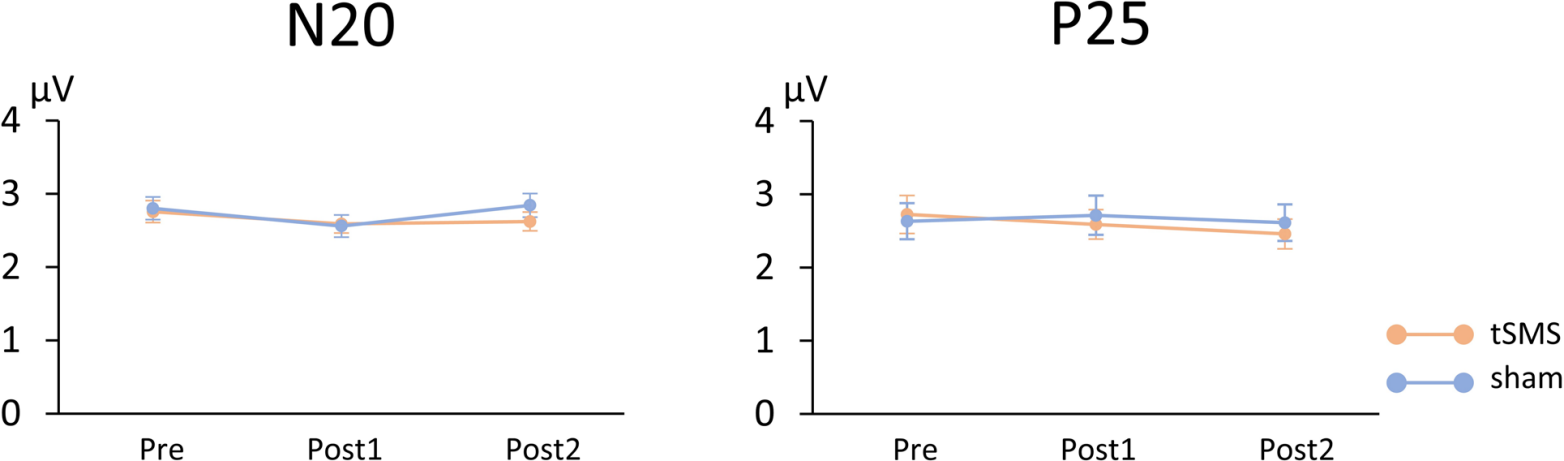
N20 and P25 amplitudes. Mean amplitudes of N20 and P25 components of somatosensory evoked potentials (SEPs) recorded before (Pre), immediately after (Post1), and 20 min after (Post2) tSMS or sham stimulation. Error bars represent the standard error of the mean.

Figure 3 (left) shows the results for the N20 amplitude. A two-way repeated-measures ANOVA revealed no significant main effect of stimulation (F(1, 19) = 0.487, *p* = 0.494) and no significant interaction between stimulation and time (F(2, 38) = 1.148, *p* = 0.328), but a significant main effect of time (F(2, 38) = 7.939, *p* = 0.001). To further examine the effect of time within each stimulation condition, planned comparisons were performed using a one-way repeated-measures ANOVA. A significant main effect of time was observed in the Sham stimulation condition (F(2, 38) = 4.227, *p* = 0.022), but not in the tSMS condition (F(2, 38) = 1.768, *p* = 0.184). However, post hoc pair-wise comparisons revealed no significant differences in N20 amplitude across time points (*p* > 0.05).

### P25 amplitude

Figure 3 (right) shows the results for the P25 amplitude. A two-way repeated-measures ANOVA revealed no significant main effect of stimulation (F(1, 19) = 0.145, *p* = 0.708) and no significant interaction between stimulation and time (F(2, 38) = 1.059, *p* = 0.357), but a significant main effect of time (F(2, 38) = 3.626, *p* = 0.036). Consistent with the results for N20 amplitude, planned comparisons were performed within each stimulation condition using a one-way repeated-measures ANOVA. This analysis showed no significant main effect of time in either the tSMS condition (F(2, 38) = 2.867, *p* = 0.069) or the Sham stimulation condition (F(2, 38) = 0.436, *p* = 0.650).

### eHFOs

**Fig. 4:**
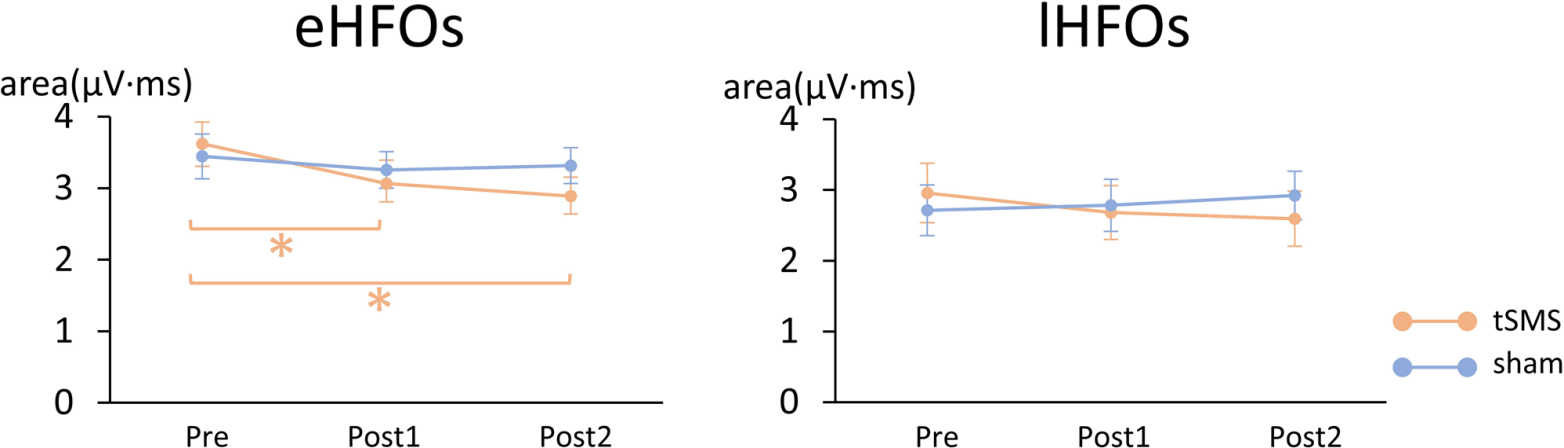
Amplitudes of high-frequency oscillations. Mean amplitudes of early and late high-frequency oscillations (eHFOs and lHFOs) recorded before (Pre), immediately after (Post1), and 20 min after (Post2) tSMS or sham stimulation. Error bars represent the standard error of the mean.

Figure 4 (left) shows the results for eHFOs amplitude. A two-way repeated-measures ANOVA revealed no significant main effect of stimulation (F(1, 19) = 0.690, *p* = 0.417) and no significant interaction between stimulation and time (F(2, 38) = 1.699, *p* = 0.196), but a significant main effect of time (F(2, 38) = 9.521, *p* < 0.001). To further explore this effect, planned comparisons were conducted within each stimulation condition using a one-way repeated-measures ANOVA. The analysis revealed a significant main effect of time in the tSMS condition (F(2, 38) = 8.311, *p* = 0.001), but not in the Sham stimulation condition (F(2, 38) = 0.446, *p* = 0.644). Post hoc pairwise comparisons indicated that eHFO amplitude significantly decreased both immediately after stimulation (*p* = 0.026) and 20 min after stimulation (*p* = 0.001) compared to baseline under the tSMS condition.

### lHFOs

Figure 4 (right) shows the results for lHFOs amplitude. A two-way repeated-measures ANOVA revealed no significant main effects of stimulation (F(1, 19) = 0.112, *p* = 0.741) and time (F(2, 38) = 0.390, *p* = 0.680), or their interaction (F(2, 38) = 1.773, *p* = 0.184).

## Discussion

The aim of this study was to explore the effects of tSMS over the S1 on low-frequency SEP components (N20 and P25) and high-frequency components (somatosensory HFOs). Our results demonstrated that tSMS modulates these SEP components in distinct ways. Specifically, as hypothesized, tSMS significantly reduced the amplitude of eHFOs, whereas neither the N20 nor lHFOs were affected. These findings provide new insights into the mechanisms through which tSMS influences cortical processing in the S1.

While the effects of tSMS on human cortical excitability and function have been demonstrated in a relatively large number of studies, the underlying mechanisms remain debated, with several hypotheses proposed in the literature. At the cellular level, these include: (i) the diamagnetic anisotropic properties of membrane phospholipids, which may induce reorientation of phospholipid molecules under static magnetic fields (SMFs)^32^, (ii) membrane surface tension generated by magnetic pressure^33^, and (iii) Lorentz force acting on ions within neuronal membrane channels^34^.

In the present study, neither the N20 amplitude nor the P25 amplitude showed significant modulation following tSMS. This lack of modulation in the N20 amplitude aligns with the findings of Carrasco-López et al. (2017)^22^ but contrasts with those of Kirimoto et al. (2014)^20^ who reported a significant reduction after applying tSMS over the S1. One possible explanation for this discrepancy is that the modulatory effects of tSMS are relatively weak and may not be sufficient to reliably alter such a stable marker as the N20 component, which reflects the activity of area 3b in the S1 driven by the thalamocortical afferent volley^35^. Indeed, short-latency components of the SEP are known to be highly stable and reproducible under standard experimental conditions and are minimally affected by physiological states such as wakefulness, light sleep, or even light anesthesia^36^. Another possible explanation concerns stimulation duration. While tSMS was applied for 20 min in both Carrasco-López et al. (2017)^22^ and the present study, Kirimoto et al. (2014)^20^ applied it for 10 and 15 min. Although this possibility remains speculative, previous studies have reported that effects of some NIBS protocols can be sensitive to stimulation duration, with extended stimulation sometimes reducing or abolishing the effect^37^. The absence of significant modulation in N20 observed here highlights the need for further research to clarify the factors shaping tSMS effects on early somatosensory processing.

We found that 20 min of tSMS attenuated eHFOs, which are thought to reflect the action potentials of thalamocortical fibers as they reach the S1^23^. The underlying mechanism of this effect may involve modulation of ion channels in thalamocortical fibers by tSMS. Specifically, as mentioned earlier, SMFs can induce reorientation of phospholipid molecules in the cell membrane due to their diamagnetic anisotropy, which may in turn alter the gating properties of calcium and sodium channels embedded in the cell membrane^32^. More recently, SMFs have also been reported to alter the activity of chloride channels^38^. Therefore, it is possible that tSMS over the S1 induces membrane deformation and voltage-sensor displacement in ion channels, ultimately suppressing action potentials of thalamocortical fibers. An important question here is why the N20 amplitude was unaffected by tSMS, whereas eHFOs were attenuated. Evidence from other inhibitory NIBS protocols provides some context. Several studies applying low-frequency rTMS or cTBS to the S1 have reported no modulation of the N20, while consistently showing changes in somatosensory HFOs^25,27,39^. In contrast, a few studies using cathodal tDCS or cTBS demonstrated a reduction in N20 amplitude^26,40^. Collectively, these findings suggest that somatosensory HFOs may represent a more sensitive marker of adaptive changes induced by inhibitory stimulation, including tSMS, whereas the N20 appears to be relatively resistant to such modulation.

In contrast to the eHFOs, tSMS produced no significant effects on the lHFOs, which reflect the activity of GABAergic interneurons within area 3b of the S1. This finding contrasts with previous studies using other inhibitory NIBS protocols, such as low-frequency rTMS and cTBS over the S1, both of which have consistently been shown to attenuate lHFOs^27,39,40^. In the M1, cTBS has been reported to reduce short-interval intracortical inhibition (SICI)^41^, a TMS paired-pulse measure mediated by GABAergic interneurons. By contrast, the effects of tSMS on SICI have been inconsistent, with some reporting enhanced SICI^12,28^ and others reporting reduced SICI^12^. Taken together, these findings suggest that, unlike cTBS^27^, tSMS may exert limited or variable effects on GABAergic interneuron activity. Consistent with this interpretation, the present results indicate that tSMS does not modulate GABAergic interneuron–related activity, at least within area 3b of the S1. Nonetheless, further studies are warranted to more fully elucidate the effects of tSMS on GABAergic function.

There are several limitations that should be acknowledged. First, all participants were healthy young adults; therefore, the present findings cannot be generalized to clinical populations. Future studies are needed to determine whether similar effects of tSMS can be observed in patient populations for whom modulation of somatosensory processing may have therapeutic relevance. Second, the present study employed a single stimulation duration and magnetic field strength. Thus, the extent to which the observed effects depend on stimulation parameters remain unclear. Systematic investigations using different stimulation durations and magnetic field intensities are warranted to determine potential dose-dependent effects and to better characterize the optimal conditions under which tSMS modulates somatosensory processing.

In conclusion, we found that tSMS applied over the S1 reduced the amplitude of eHFOs but not that of lHFOs, N20, or P25. These findings suggest that tSMS selectively suppresses thalamocortical input to area 3b, while leaving neural activity within area 3b of the S1 and the activity of local GABAergic interneurons largely unaffected. This study provides novel insights into the physiological mechanisms underlying the modulatory effects of tSMS on somatosensory cortical processing.

## Supplementary Information

Below is the link to the electronic supplementary material.

Supplementary Material 1

## Data Availability

The datasets used during the study will be available from the corresponding author on reasonable request.

## References

[1] Oliviero, A. et al. Transcranial static magnetic field stimulation of the human motor cortex. *J. Physiol.* **589**, 4949–4958. 10.1113/jphysiol.2011.211953 (2011). 21807616 10.1113/jphysiol.2011.211953 PMC3224885

[2] Kirimoto, H., Asao, A., Tamaki, H. & Onishi, H. Non-invasive modulation of somatosensory evoked potentials by the application of static magnetic fields over the primary and supplementary motor cortices. *Sci. Rep.* **6**, 34509. 10.1038/srep34509 (2016). 27698365 10.1038/srep34509 PMC5048290

[3] Oliviero, A. et al. Safety study of transcranial static magnetic field stimulation (tSMS) of the human cortex. *Brain Stimul*. **8**, 481–485. 10.1016/j.brs.2014.12.002 (2015). 25595064 10.1016/j.brs.2014.12.002

[4] Kirimoto, H. et al. Transcranial static magnetic field stimulation over the primary motor cortex induces plastic changes in cortical nociceptive processing. *Front. Hum. Neurosci.* **12**, 63. 10.3389/fnhum.2018.00063 (2018). 29497371 10.3389/fnhum.2018.00063 PMC5818436

[5] Watanabe, T. et al. Null effect of transcranial static magnetic field stimulation over the dorsolateral prefrontal cortex on behavioral performance in a Go/NoGo task. *Brain Sci.* **11**10.3390/brainsci11040483 (2021). 10.3390/brainsci11040483 PMC8069672 33920398

[6] Chen, X. et al. Transient modulation of working memory performance and Event-Related potentials by transcranial static magnetic field stimulation over the dorsolateral prefrontal cortex. *Brain Sci.* **11**10.3390/brainsci11060739 (2021). 10.3390/brainsci11060739 PMC8228367 34199505

[7] Tsuru, D. et al. The effects of transcranial static magnetic fields stimulation over the supplementary motor area on anticipatory postural adjustments. *Neurosci. Lett.* **723**, 134863. 10.1016/j.neulet.2020.134863 (2020). 32105767 10.1016/j.neulet.2020.134863

[8] Azcona Ganuza, G. & Alegre, M. Static magnetic stimulation of human auditory cortex: a feasibility study. *Neuroreport* **33**, 487–494. 10.1097/WNR.0000000000001809 (2022). 35767229 10.1097/WNR.0000000000001809 PMC9245555

[9] Kirimoto, H. et al. Influence of static magnetic field stimulation on the accuracy of tachystoscopically presented line bisection. *Brain Sci.* **10**10.3390/brainsci10121006 (2020). 10.3390/brainsci10121006 PMC7766566 33352946

[10] Gonzalez-Rosa, J. J. et al. Static magnetic field stimulation over the visual cortex increases alpha oscillations and slows visual search in humans. *J. Neurosci.* **35**, 9182–9193. 10.1523/JNEUROSCI.4232-14.2015 (2015). 26085640 10.1523/JNEUROSCI.4232-14.2015 PMC6605156

[11] Matsugi, A. & Okada, Y. Cerebellar transcranial static magnetic field stimulation transiently reduces cerebellar brain Inhibition. *Funct. Neurol.* **32**, 77–82. 10.11138/fneur/2017.32.2.077 (2017). 28676140 10.11138/FNeur/2017.32.2.077 PMC5507156

[12] Dileone, M., Mordillo-Mateos, L., Oliviero, A. & Foffani, G. Long-lasting effects of transcranial static magnetic field stimulation on motor cortex excitability. *Brain Stimul*. **11**, 676–688. 10.1016/j.brs.2018.02.005 (2018). 29500043 10.1016/j.brs.2018.02.005

[13] Shibata, S. et al. Effect of transcranial static magnetic stimulation on intracortical excitability in the contralateral primary motor cortex. *Neurosci. Lett.* **723**, 134871. 10.1016/j.neulet.2020.134871 (2020). 32109553 10.1016/j.neulet.2020.134871

[14] Takamatsu, Y. et al. Transcranial static magnetic stimulation over the motor cortex can facilitate the contralateral cortical excitability in human. *Sci. Rep.* **11**, 5370. 10.1038/s41598-021-84823-4 (2021). 33686102 10.1038/s41598-021-84823-4 PMC7940605

[15] Dileone, M. et al. Home-based transcranial static magnetic field stimulation of the motor cortex for treating levodopa-induced dyskinesias in parkinson’s disease: A randomized controlled trial. *Brain Stimul*. **15**, 857–860. 10.1016/j.brs.2022.05.012 (2022). 35609815 10.1016/j.brs.2022.05.012

[16] Di Lazzaro, V. et al. Transcranial static magnetic stimulation for amyotrophic lateral sclerosis: a bicentric, randomised, double-blind placebo-controlled phase 2 trial. *Lancet Reg. Health Eur.* **45**, 101019. 10.1016/j.lanepe.2024.101019 (2024). 39185360 10.1016/j.lanepe.2024.101019 PMC11341967

[17] Shimomura, R. et al. Transcranial static magnetic field stimulation (tSMS) can induce functional recovery in patients with subacute stroke. *Brain Stimul*. **16**, 933–935. 10.1016/j.brs.2023.05.024 (2023). 37257816 10.1016/j.brs.2023.05.024

[18] Yamada, T., Yeh, M. & Kimura, J. Fundamental principles of somatosensory evoked potentials. *Phys. Med. Rehabil Clin. N Am.* **15**, 19–42. 10.1016/s1047-9651(03)00100-1 (2004). 15029897 10.1016/s1047-9651(03)00100-1

[19] Fustes, O. J. H. et al. Somatosensory evoked potentials in clinical practice: a review. *Arq. Neuropsiquiatr.* **79**, 824–831. 10.1590/0004-282X-ANP-2020-0427 (2021). 34669817 10.1590/0004-282X-ANP-2020-0427

[20] Kirimoto, H. et al. Effect of transcranial static magnetic field stimulation over the sensorimotor cortex on somatosensory evoked potentials in humans. *Brain Stimul*. **7**, 836–840. 10.1016/j.brs.2014.09.016 (2014). 25444588 10.1016/j.brs.2014.09.016

[21] Wood, C. C., Cohen, D., Cuffin, B. N., Yarita, M. & Allison, T. Electrical sources in human somatosensory cortex: identification by combined magnetic and potential recordings. *Science* **227**, 1051–1053. 10.1126/science.3975600 (1985). 3975600 10.1126/science.3975600

[22] Carrasco-Lopez, C. et al. Static magnetic field stimulation over parietal cortex enhances somatosensory detection in humans. *J. Neurosci.* **37**, 3840–3847. 10.1523/JNEUROSCI.2123-16.2017 (2017). 28280254 10.1523/JNEUROSCI.2123-16.2017 PMC6596712

[23] Ozaki, I. & Hashimoto, I. Exploring the physiology and function of high-frequency oscillations (HFOs) from the somatosensory cortex. *Clin. Neurophysiol.* **122**, 1908–1923. 10.1016/j.clinph.2011.05.023 (2011). 21724458 10.1016/j.clinph.2011.05.023

[24] Gobbele, R. et al. Different origins of low- and high-frequency components (600 Hz) of human somatosensory evoked potentials. *Clin. Neurophysiol.* **115**, 927–937. 10.1016/j.clinph.2003.11.009 (2004). 15003775 10.1016/j.clinph.2003.11.009

[25] Ogawa, A. et al. Slow repetitive transcranial magnetic stimulation increases somatosensory high-frequency oscillations in humans. *Neurosci. Lett.* **358**, 193–196. 10.1016/j.neulet.2004.01.038 (2004). 15039114 10.1016/j.neulet.2004.01.038

[26] Dieckhofer, A. et al. Transcranial direct current stimulation applied over the somatosensory cortex - differential effect on low and high frequency SEPs. *Clin. Neurophysiol.* **117**, 2221–2227. 10.1016/j.clinph.2006.07.136 (2006). 16931142 10.1016/j.clinph.2006.07.136

[27] Katayama, T., Suppa, A. & Rothwell, J. C. Somatosensory evoked potentials and high frequency oscillations are differently modulated by theta burst stimulation over primary somatosensory cortex in humans. *Clin. Neurophysiol.* **121**, 2097–2103. 10.1016/j.clinph.2010.05.014 (2010). 20554474 10.1016/j.clinph.2010.05.014

[28] Nojima, I., Koganemaru, S., Fukuyama, H. & Mima, T. Static magnetic field can transiently alter the human intracortical inhibitory system. *Clin. Neurophysiol.* **126**, 2314–2319. 10.1016/j.clinph.2015.01.030 (2015). 25792074 10.1016/j.clinph.2015.01.030

[29] Arias, P., Adan-Arcay, L., Puerta-Catoira, B., Madrid, A. & Cudeiro, J. Transcranial static magnetic field stimulation of M1 reduces corticospinal excitability without distorting sensorimotor integration in humans. *Brain Stimul*. **10**, 340–342. 10.1016/j.brs.2017.01.002 (2017). 28094125 10.1016/j.brs.2017.01.002

[30] Davila-Perez, P., Pascual-Leone, A. & Cudeiro, J. Effects of transcranial static magnetic stimulation on motor cortex evaluated by different TMS waveforms and current directions. *Neuroscience* **413**, 22–30. 10.1016/j.neuroscience.2019.05.065 (2019). 31195056 10.1016/j.neuroscience.2019.05.065 PMC6688472

[31] Ozaki, I. et al. High frequency oscillations in early cortical somatosensory evoked potentials. *Electroencephalogr. Clin. Neurophysiol.* **108**, 536–542. 10.1016/s0168-5597(98)00032-x (1998). 9872424 10.1016/s0168-5597(98)00032-x

[32] Rosen, A. D. Mechanism of action of moderate-intensity static magnetic fields on biological systems. *Cell. Biochem. Biophys.* **39**, 163–173. 10.1385/CBB:39:2:163 (2003). 14515021 10.1385/CBB:39:2:163

[33] Hernando, A. et al. Effects of moderate static magnetic field on neural systems is a Non-invasive mechanical stimulation of the brain possible theoretically? *Front. Neurosci.* **14**, 419. 10.3389/fnins.2020.00419 (2020). 32508563 10.3389/fnins.2020.00419 PMC7248270

[34] Freire, M. J., Bernal-Mendez, J. & Perez, A. T. The Lorentz force on ions in membrane channels of neurons as a mechanism for transcranial static magnetic stimulation. *Electromagn. Biol. Med.* **39**, 310–315. 10.1080/15368378.2020.1793172 (2020). 32666841 10.1080/15368378.2020.1793172

[35] Allison, T., McCarthy, G., Wood, C. C. & Jones, S. J. Potentials evoked in human and monkey cerebral cortex by stimulation of the median nerve. A review of scalp and intracranial recordings. *Brain* **114** (Pt 6), 2465–2503. 10.1093/brain/114.6.2465 (1991). 1782527 10.1093/brain/114.6.2465

[36] Eisen, A. The somatosensory evoked potential. *Can. J. Neurol. Sci.* **9**, 65–77. 10.1017/s0317167100043717 (1982). 6286081 10.1017/s0317167100043717

[37] Vignaud, P., Mondino, M., Poulet, E., Palm, U. & Brunelin, J. Duration but not intensity influences transcranial direct current stimulation (tDCS) after-effects on cortical excitability. *Neurophysiol. Clin.* **48**, 89–92. 10.1016/j.neucli.2018.02.001 (2018). 29482881 10.1016/j.neucli.2018.02.001

[38] Sinha, A. S. et al. Static magnetic field stimulation enhances shunting Inhibition via a SLC26 family Cl(-) Channel, inducing intrinsic plasticity. *J. Neurosci.* **44**10.1523/JNEUROSCI.1324-22.2024 (2024). 10.1523/JNEUROSCI.1324-22.2024 PMC10904086 38302440

[39] Restuccia, D., Ulivelli, M., De Capua, A., Bartalini, S. & Rossi, S. Modulation of high-frequency (600 Hz) somatosensory-evoked potentials after rTMS of the primary sensory cortex. *Eur. J. Neurosci.* **26**, 2349–2358. 10.1111/j.1460-9568.2007.05828.x (2007). 17894818 10.1111/j.1460-9568.2007.05828.x

[40] Rocchi, L., Casula, E., Tocco, P., Berardelli, A. & Rothwell, J. Somatosensory Temporal discrimination threshold involves inhibitory mechanisms in the primary somatosensory area. *J. Neurosci.* **36**, 325–335. 10.1523/JNEUROSCI.2008-15.2016 (2016). 26758826 10.1523/JNEUROSCI.2008-15.2016 PMC6602023

[41] Murakami, T., Sakuma, K., Nomura, T., Nakashima, K. & Hashimoto, I. High-frequency oscillations change in parallel with short-interval intracortical Inhibition after theta burst magnetic stimulation. *Clin. Neurophysiol.* **119**, 301–308. 10.1016/j.clinph.2007.10.012 (2008). 18063408 10.1016/j.clinph.2007.10.012

